# The association between fatigue severity and risk of falls among middle-aged and older Australian stroke survivors

**DOI:** 10.1007/s40520-022-02179-9

**Published:** 2022-07-07

**Authors:** David Sibbritt, Jessica Bayes, Wenbo Peng, Jane Maguire, Suzy Ladanyi, Jon Adams

**Affiliations:** 1 School of Public Health, Faculty of Health, University of Technology Sydney, 15 Broadway, Ultimo, NSW 2007 Australia; 2 School of Nursing and Midwifery, Faculty of Health, University of Technology Sydney, Ultimo, NSW Australia

**Keywords:** Stroke, Fatigue, Falls, Rehabilitation

## Abstract

**Background:**

Fatigue is a common and often debilitating symptom experienced by many stroke survivors. Significant post stroke fatigue may predispose individuals to other health complications, such as falls, which can lead to fractures and soft tissue injuries. Only limited research has examined the association between fatigue and falls in stroke survivors.

**Methods:**

Data were obtained from the Sax Institute’s 45 and Up Study, from a subset of individuals who had experienced a stroke. The Modified Fatigue Impact Scale—5-item version (MFIS-5) was used to measure the level of fatigue. A logistic regression model, adjusted for stroke characteristics and comorbidities, was used to determine the magnitude of association between change in fatigue score and odds of having had a fall.

**Results:**

A total of 576 participants completed the questionnaire. A total of 214 (37.2%) participants reported having had a fall in the previous 12 months. There was a statistically significant association between fatigue scores and fall status (*p* < 0.001). Specifically, for every 1-point increase in the fatigue score (MFIS-5) (i.e. higher level of fatigue), the odds of a person having a fall is 1.10 times greater (AOR = 1.10; 95% CI 1.05, 1.15; *p* < 0.001).

**Conclusion:**

This study revealed an association between an increasing risk of falls with increasing severity of post stroke fatigue. Accurate detection and management of fatigue may help reduce the risk of falls and should be the focus of future research.

## Introduction

The term *stroke* is typically used to describe a neurological injury to the central nervous system (CNS) by a vascular cause which can be divided into: cerebral infarction; intracerebral haemorrhage (ICH); and subarachnoid haemorrhage (SAH) [1]. Stroke is a major source of morbidity and mortality and is the second leading cause of death and second most common cause of disability adjusted life years (DALY’s) worldwide [2]. Research predicts a rise in the prevalence of stroke, based on evidence of an aging population in countries such as Australia [3]. This could lead to an increased burden across all areas of stroke care, from acute management to rehabilitation and long-term care [3].

Recent advances in stroke management and outcomes have concentrated on hyper-acute and acute care while the post-acute phase has received much less attention [4]. It is estimated that over 30 million stroke survivors are living with post-stroke disability worldwide [5]. Recent research found that one in five people live at least 15 years after a stroke and concluded that poor functional, cognitive and psychological outcomes affect a substantial proportion of these long-term survivors [6]. Common comorbidities among stroke survivors include pain, depression, and fatigue which can significantly affect health related quality of life [7].

Fatigue is common and often debilitating with 40% of stroke patients affected by fatigue [7]. Physical and cognitive fatigue can impact gait control [8], impair balance and limit mobility, which can increase the risk of falls [9]. Post-stroke fatigue can adversely affect daily activities, the ability to return to work, and the participation in rehabilitation programmes [10]. Many factors are thought to contribute to post stroke fatigue, including physical factors such as an increased level of disability, neurophysiological factors such as corticomotor excitability, and psychological factors such as depression and anxiety [10]. Inflammation is also thought to play a significant role, with research demonstrating that inflammatory cytokines are upregulated in the brain after stroke [11]. Specifically, IL-1β appears to be a predictor of post stroke fatigue [12] with researchers suggesting that fatigue after stroke may be due to inflammation-induced sickness behaviour [11]. Significant post stroke fatigue may predispose stroke survivors to other health complications, such as falls.

Falls among stroke survivors are common, with 40–58% of individuals experiencing a fall within 1 year of their stroke [13–15]. Falls can lead to further physical health complications such as fractures [16], soft tissue injuries and impairment to functional activities [17]. Additionally, if a hip fracture is sustained as a result of the fall, stroke survivors are less likely to regain independent mobility than the general population [18]. Consequently, stroke survivors often develop psychosocial issues after a fall such as depression [19] and a fear of falling again [20]. This can lead to individuals reducing their level of activity further, causing more physical deconditioning and, loss of independence, resulting in poorer quality of life [17].

To date, only limited research has examined the association between fatigue and falls in stroke survivors. A qualitative review which examined the experiences of recurrent fallers in the first year after stroke reported that participants view themselves at higher risk of falls when they feel tired [21]. However, a small scale observational case–control study which included a fatigue rating scale found no association between fatigue and falls [20]. More research is needed to explore if a relationship between fatigue and risk of falling exists in stroke survivors. The purpose of this research is to address this research gap with the hope of identifying risk factors and therapeutic targets for stroke care and management.

## Methods

### Sample

Data were obtained from a sub-study of the Sax Institute’s 45 and Up Study, which is conducted in Australia[22]. The baseline questionnaire collected information from 267,153 men and women aged 45 and above who resided in the state of New South Wales, Australia. Participants were randomly sampled from the Services Australia (formerly the Australian Government Department of Human Services) Medicare enrolment database. The sample represented approximately 11% of the NSW population aged 45 years and over with a response rate of about 18%. The 45 and Up Study was approved by the University of New South Wales Human Research Ethics Committee. People aged 80+ years and residents of rural and remote areas were oversampled. Participants joined the Study by completing a baseline questionnaire between January 2006 and December 2009. The sub-study survey of participants from this cohort occurred between April and October 2017. For this sub-study, 1,300 participants who had previously indicated on the baseline questionnaire that a doctor had diagnosed them as having had a stroke were mailed a sub-study questionnaire, with 576 (44.3% response) returning a completed questionnaire.

### Demographic measures

Demographic measures included age, gender, marital status and education. Participants were also asked how they managed on their available income. In addition, area of residence was defined using the Accessibility/Remoteness Index of Australia Plus (ARIA+) remoteness score, which uses postcode to determine road distances to service centres, and thus participants were categorised as residing in a major city, inner regional area, or outer regional/remote area [23].

### Stroke-related measures

Participants were asked to specify the time (years/months) since they were first diagnosed with stroke and also the number of strokes they had experienced. The Modified Fatigue Impact Scale—5-item version (MFIS-5) was used to measure the level of fatigue[24]. The total score ranges from 0 to 20, with a higher score indicating more severe fatigue. A MFIS-5 score of > 10 is considered high fatigue[25]. The participants were asked to rate the degree of disability or dependence in the daily activities using the modified Rankin Scale (mRS)[26].

### Health status measures

The survey collected self‐reported measures of height and weight, which were used to calculate body mass index (BMI). BMI was used to categorised participants according to WHO recommendations: underweight as BMI < 18.5 kg m^2^; healthy weight as BMI from 18.5 to 24.99 kg m^2^; overweight as BMI from 25 to 29.99 kg m^2^; and obese as BMI ≥ 30 kg m^2^ [27]. Participants were asked about their consumption of alcohol. Based on National Health and Medical Research Council (NHMRC) guidelines, a variable for alcohol status was derived from the frequency and quantity of alcohol consumption, comprising of 4 categories: ‘non-drinkers’; ‘low risk’ (up to14 drinks per week); ‘risky’ (15–28 drinks per week); and ‘high risk’ (more than 28 drinks per week) [28].

In addition, participants were asked about their smoking status, which was defined as either non-smoking (i.e. never smoked or not smoking now but have smoked in the past) or smoking. Participants were also asked to report the number of times as well as the time (hours and minutes) spent in the last week: walking briskly, in moderate intensity leisure activities (e.g. social tennis, recreational swimming), in vigorous leisure activity (e.g. competitive sport, running), or vigorous household chores (i.e. that make you breathe harder or puff and pant). Responses to these questions were summed and assigned a metabolic equivalent (MET) value. Based on the MET value, participants were categorised to indicate the overall volume of physical activity, as followed: 0 to < 500 (inactive or low); 500 to < 1000 (moderately active); ≥ 1000 (highly active) [29].

The participants were asked if they had been diagnosed or treated by a doctor for any of the following conditions: anxiety/nervous disorder, asthma, cancer, dementia/Alzheimer’s disease, depression, diabetes, heart disease, hypertension, osteoarthritis, and/or osteoporosis. Participants were also asked if they had taken or used any prescription medications, other than those for their stroke, during the past 12 months. These prescription medications were categorised into psychotropic (e.g. Avanza, Epilim, Gabapentin) or non-psychotropic.

### Statistical analyses

Chi-square tests were used to examine the association between categorical variables. These included depression status, demographic factors, health status, health care utilisation, and self-care activity measures.. A multiple logistic regression model was then used to determine the magnitude of association between change in fatigue score and odds of having had a fall. Note that this regression model also included potential confounding variables, as determined by the bivariate analyses (i.e. statistically significant demographic and health status characteristics). All analyses were conducted using the statistical software Stata, version 14.1. Statistical significance was set at the *α* = 0.05 level.

## Results

The average age of participants was 75.8 (SD = 9.1) years, with 54.9% being male and 45.1% female. The majority of participants resided in a major city (52.2%), followed by an inner regional area (33.9%) and outer regional or remote area (13.9%).

The associations between stroke characteristics and having had a fall in the previous 12 months are presented in Table 1. It can be seen that a participant was more likely to have had a fall if they had had three or more strokes, compare to those who only had one stroke (*p* = 0.035). Participants with a high degree of stroke disability were more likely to have had a fall, compared to those with a low degree of disability (*p* = 0.012). There were 60 (10.4%) participants considered to have high fatigue (MFIS-5 > 10) and there was a statistically significant difference in fatigue scores between those participants who had a fall (mean = 5.5) and those participants who did not have a fall (mean = 3.4) (*p* < 0.001).

**Table 1:** The association between stroke characteristics and having had a fall in the previous 12 months

Stroke characteristics	Total	Had a fall		*p* value
		Yes	No	
		*n* (%)	*n* (%)	
Number of strokes				
1	428	152 (71.0)	276 (76.3)	0.035
2	89	31 (14.5)	58 (16.0)	
3+	59	31 (14.5)	28 (7.7)	
Degree of disability mRS^a^				
Low (0–2 points)	374	125 (58.4)	249 (68.8)	0.012
High (3–5 points)	202	89 (41.6)	113 (31.2)	

		Mean (SD)	Mean (SD)	Mean (SD)	
Stroke duration	Years since most recent stoke	10.4 (8.9)	10.5 (8.7)	10.4 (9.1)	0.968
Fatigue	MFIS-5^b^	4.2 (4.2)	5.5 (4.4)	3.4 (3.9)	< 0.001

^a^Modified Rankin Scale (mRS) (0 = no disability and 5 = severe disability)

^b^Modified Fatigue Impact Scale—5-item version (MFIS-5)

Overall, 214 (37.2%) participants reported having had a fall in the previous 12 months. Details of these falls were as follows: 207 (35.9%) had a fall to the ground; 104 (18.1%) had been injured as a result of a fall; and 89 (15.5%) needed to seek medical attention for an injury from a fall. Table 2 shows the associations between demographic characteristics and having had a fall in the previous 12 months. It can be seen that none of the demographic characteristics were statistically significant associated with having had a fall.

**Table 2:** The association between demographic characteristics and having had a fall in the previous 12 months

Demographic characteristics	Total	Had a fall		*p* value
		Yes	No	
		*n* (%)	*n* (%)	
Gender				
Male	316	126 (58.9)	190 (52.5)	0.136
Female	260	88 (41.1)	172 (47.5)	
Area of residence^A^				
Major cities	296	108 (51.2)	188 (52.8)	0.797
Inner regional	192	75 (35.6)	117 (32.9)	
Outer regional or remote	79	28 (13.3)	51 (14.3)	
Marital status^B^				
Married or defacto	359	128 (61.0)	231 (64.7)	0.626
Separated or divorced or widowed	157	63 (30.0)	94 (26.3)	
Single	51	19 (9.0)	32 (9.0)	
Educational qualification^C^				
No formal school or school only	276	109 (51.7)	167 (46.4)	0.135
Trade or apprentice or diploma	187	71 (33.7)	116 (32.2)	
University	108	31 (14.6)	77 (21.4)	

		Mean (SD)	Mean (SD)	Mean (SD)	
Age (in years)		75.8 (9.1)	76.8 (9.4)	75.3 (8.9)	0.054

^A^Missing area of residence data for 9 participants (6 who did not have a fall and 3 who did have a fall)

^B^Missing marital status data for 9 participants (5 who did not have a fall and 3 who did have a fall)

^C^Missing educational qualification data for 5 participants (2 who did not have a fall and 3 who did have a fall)

Table 3 shows the association between health status and having had a fall in the previous 12 months. It can be seen that participants with anxiety or nervous disorder were more likely to have had a fall, compared to those without anxiety or nervous disorder (*p* = 0.001). Participants with depression were more likely to have had a fall, compared to those without depression (*p* < 0.001). Participants with diabetes were more likely to have had a fall, compared to those without diabetes (*p* < 0.001). Participants with osteoarthritis were more likely to have had a fall, compared to those without osteoarthritis (*p* = 0.022). In addition, those participants who were taking a psychotropic medication were more likely to have had a fall, compared to those who were not taking a psychotropic medication (*p* = 0.001).

**Table 3:** The association between health status and having had a fall in the previous 12 months

Health status characteristics	Total	Had a fall		*p* value
		Yes	No	
		*n* (%)	*n* (%)	
Anxiety/nervous disorder				
No	524	184 (86.0)	340 (93.9)	0.001
Yes	52	30 (14.0)	22 (6.1)	
Asthma				
No	521	191 (89.3)	330 (91.2)	0.452
Yes	55	23 (10.7)	32 (8.84)	
Cancer				
No	528	191 (89.3)	337 (93.1)	0.107
Yes	48	23 (10.7)	25 (6.9)	
Dementia/Alzheimer’s				
No	568	210 (98.1)	358 (98.9)	0.449
Yes	8	4 (1.9)	4 (1.1)	
Depression				
No	519	179 (83.6)	340 (93.9)	< 0.001
Yes	57	35 (16.4)	22 (6.1)	
Diabetes				
No	474	161 (75.2)	313 (86.5)	< 0.001
Yes	102	53 (24.8)	49 (13.5)	
Heart disease				
No	438	156 (72.9)	282 (77.9)	0.174
Yes	138	58 (27.1)	80 (22.1)	
Hypertension				
No	374	136 (63.6)	238 (65.8)	0.594
Yes	202	78 (36.4)	124 (34.2)	
Osteoarthritis				
No	437	151 (70.6)	286 (79.0)	0.022
Yes	139	63 (29.4)	76 (21.0)	
Osteoporosis				
No	514	190 (88.8)	324 (89.5)	0.788
Yes	62	24 (11.2)	38 (10.5)	
Psychotropic medication				
No	555	199 (93.0)	356 (98.3)	0.001
Yes	21	15 (7.0)	6 (1.7)	
Alcohol consumption^A^				
No or low risk	480	178 (84.0)	302 (84.4)	0.900
Risky or high risk	90	34 (16.0)	56 (15.6)	
Smoking status^B^				
Non-smoker	490	179 (87.8)	311 (90.7)	0.279
Current smoker	57	25 (12.3)	32 (9.3)	
Physical activity^C^				
Inactive or sedentary	204	85 (43.2)	119 (35.3)	0.198
Moderately active	89	30 (15.2)	59 (17.5)	
Highly active	241	82 (41.6)	159 (47.2)	
BMI category^D^				
Normal or underweight	180	65 (34.6)	115 (35.9)	0.100
Overweight	195	64 (34.0)	131 (40.9)	
Obese	133	59 (31.4)	74 (23.2)	

^A^Missing alcohol consumption data for 6 participants (4 who did not have a fall and 2 who did have a fall)

^B^Missing smoking status data for 29 participants (19 who did not have a fall and 10 who did have a fall)

^C^Missing physical activity data for 42 participants (25 who did not have a fall and 17 who did have a fall)

^D^Missing BMI category data for 68 participants (42 who did not have a fall and 26 who did have a fall)

A logistic regression model was used to determine the magnitude of association between change in fatigue score and odds of having had a fall. The subsequent odds ratio was adjusted for all variables identified as being statistically significantly associated to falls in the bivariate analyses (Tables 1, 2, 3) (i.e. number of strokes, stroke disability, use of psychotropic medications, anxiety, depression, diabetes, osteoarthritis). The resulting model revealed that fatigue is statistically significantly associated with falls. Specifically, for every 1-point increase in the fatigue score (MFIS-5) (i.e. higher level of fatigue), the odds of a person having a fall is 1.10 times greater (AOR = 1.10; 95% CI 1.05, 1.15; *p* < 0.001).

## Discussion

Our study of middle-aged and older Australian adult stroke survivors revealed that the prevalence on falls was 37.2% and severe fatigue was 10.4%. We hypothesised that the risk of falls among stroke survivors increases with higher levels of fatigue. Our results confirm this association, with a 10% increase in risk of falls for every 1-point increase in fatigue score. Our study finding is consistent with previous research in other nervous system conditions such as multiple sclerosis (MS) [30] and Parkinson’s disease [31]. Research has shown that individuals affected by MS who indicated high fatigue levels performed significantly worse on tests of balance and had more falls in a 6-month period compared to those with low fatigue levels [30]. Similar observations have been made in patients with Parkinson’s disease where severity of fatigue was related to an increased number of falls [31].

Several explanations have been hypothesised for the association between fatigue and risk of falls in older adults including gait control, daytime sleepiness, pain, anaemia, and metabolic disorders [32]. Previous research has shown that physical fatigue also has an impact on walking and gait control in older people [8] and that impaired balance, decreased lower extremity strength, and limited mobility are common physiological risk factors for falling [9]. These are common complaints experienced by stroke survivors which may partially explain the high incidence of falls among this population.

Excessive daytime sleepiness (EDS) becomes a chronic problem in 34% of stroke survivors and can significantly affect a stroke survivor’s daytime functional performance [33]. EDS can increase the frequency of falls through several different mechanisms [34]. For example, for healthy mobilisation and balance coordination, there must be effective integration of visual, vestibular, and proprioceptive senses [35]. This requires a high level of attention which can be lacking in those suffering from EDS [35].

Other explanations postulated for the association between fatigue and falls is the presence of other underlying health conditions such as anaemia [36]. Anaemia is relatively common among stroke survivors with prevalence ranging from 15 to 30% of individuals [37]. A recent systematic review and meta-analysis demonstrated an association between anaemia and poor outcomes in stroke survivors [38]. Research has also demonstrated a relationship between falls during hospitalisation in older patients and the presence of anaemia [36]. Anaemia can lead to weakness, fatigue and limitations to activity [36] which may explain the increased risk of falls.

It is interesting to note that alcohol consumption did not affect the risk of falling in our cohort of stoke survivors. One possible explanation for this finding is that risky consumption of alcohol was low, only 15% of the sample, and as such there may be an insufficient number of risky consumers of alcohol to result in statically significant findings.

This research has highlighted the significant relationship between fatigue and falls in stroke survivors. Due to the many long-term consequences of falls in this demographic, identifying and measuring fatigue should be considered in all fall risk assessments. Management of fatigue in stoke survivors should be the focus of future research, as this may contribute to fall prevention. Unfortunately, evidence-based pharmacological treatment options for treating fatigue in neurological conditions such as stroke are scarce [39]. There are some promising non-pharmaceutical treatments, but more research is needed to determine if these treatments also reduce the risk of falls. Non-pharmacological strategies such as mindfulness-based stress reduction [40], yoga, exercise training and cognitive rehabilitation [41] have shown to be effective in treating fatigue resulting from neurological diseases. But again, more research is needed to determine if these fatigue interventions also impact falls and risk of falling.

This study has utilised widely used, validated instruments to measure key variables used in our analyses and is nested within the largest ongoing cohort study of healthy ageing in the Southern Hemisphere. However, there are some limitations which need to be taken into consideration when interpreting these findings. The cohort data are based on respondents' self-reporting and may have the potential for recall bias. Additionally, the limited information about the type and severity of stroke may impact these results. Although several studies have validated the MFIS-5 instrument in Multiple Sclerosis populations, there has been no such validation of the instrument in stroke populations. However, D’Souza [24], in her review of the MFIS-5 instrument, stated that she found it useful in her assessment of patients with conditions in which fatigue is a dominant symptom, such as stroke [24]. Note also that Larson [42] states that the MFIS-5 does have some limitations when interpreting the scores, particularly when trying to interpret what a change in score means [42].

## Conclusion

This study found a significant, positive association between risk of falls and fatigue among older Australian stroke survivors. Accurate detection and management of fatigue may help reduce the risk of falls in stoke survivors and should be the focus of future research.

## Data Availability

The data set could potentially be made available to other researchers if they obtain the necessary approvals. Further information on this process can be obtained from the 45 and Up Study (45andUp.research@ saxinstitute.org.au).
